# Determination of canine ROTEM Sigma reference ranges and comparative analysis with ROTEM Delta

**DOI:** 10.3389/fvets.2025.1737842

**Published:** 2026-01-07

**Authors:** Aurélie Jourdan, Julia Noval Montero, Geoffrey Troussier, Anna Anderco, Caroline Dania, Yann Moumadah, Maxime Cambournac

**Affiliations:** 1 Centre Hospitalier Vétérinaire Frégis, Paris, France; 2 IVC Evidensia France, Courbevoie, France

**Keywords:** clot strength, coagulation, hemostasis, reference intervals, rotational thromboelastometry, ROTEM, viscoelastic testing

## Abstract

**Introduction:**

Hemostatic dysfunctions are frequent in critically ill dogs and can markedly affect clinical outcomes. Conventional plasma-based coagulation testing provides limited insight into the complex *in vivo* interactions among platelets, red blood cells, and coagulation factors. Viscoelastic testing has emerged to overcome these limitations by assessing the dynamics of clot formation and breakdown in whole blood. Among these, ROtational ThromboElastoMetry (ROTEM) provides a real-time evaluation of the coagulation process, reflecting clot initiation, propagation, and lysis. The recently developed ROTEM Sigma analyzer automates reagent handling and measurement minimizing operator variability and pre-analytical errors. Despite its growing use in human medicine, canine reference intervals for the ROTEM Sigma platform have not yet been established.

**Objective:**

To determine the reference intervals for ROTEM Sigma parameters in healthy dogs, to compare these values with published ROTEM Delta data, and to assess the influence of physiological variables such as age, sex, body weight, and neuter status.

**Methods:**

A prospective observational study was conducted in 42 clinically healthy dogs. Whole blood samples were analyzed using ROTEM Sigma EXTEM, INTEM, FIBTEM, and HEPTEM assays. Reference intervals were generated using both conventional parametric and robust median–MAD statistical methods. Comparisons were made with previously reported canine ROTEM Delta data. Multivariate regression explored associations between ROTEM parameters and demographic or hematologic variables.

**Results:**

Reference intervals were successfully defined for all ROTEM Sigma parameters (CT, CFT, A10, MCF, LI60, and G). EXTEM values agreed most closely with previously published ROTEM Delta data, whereas INTEM and FIBTEM exhibited greater variability. Regression analysis identified maximum clot firmness (MCF) as the only parameter significantly affected by biological variables. MCF decreased with higher body weight, increased with platelet count and showed minor sex-related differences.

**Conclusion:**

This study establishes canine-specific ROTEM Sigma reference intervals. The close concordance with Delta data more (particularly EXTEM) suggests cross platform consistency while highlighting the necessity of dedicated Sigma-specific reference ranges. Body weight, sex, and platelet count were the primary determinants of MCF. These validated reference intervals enhance the clinical applicability of ROTEM Sigma for assessing hemostatic function in dogs.

## 1 Introduction

Hemostatic disorders are common in critically ill dogs, with over half of ICU patients exhibiting coagulation abnormalities (1, 2). These dysfunctions can involve any stage of the coagulation process, including primary hemostasis, secondary hemostasis, and fibrinolysis. Conventional assays, such as platelet counts, Prothrombin Time (PT), and Activated Partial Thromboplastin Time (aPTT), evaluate isolated components of hemostasis and provide limited insight into platelet function, clot dynamics, or fibrinolysis. While specialized tests such as platelet aggregometry can assess platelet function more directly, they require specialized equipment and strict pre-analytical control (3). Traditional plasma-based and reductionist assays fail to reflect the integrated nature of *in vivo* coagulation, which is governed by interactions among platelets, red and white blood cells, plasma coagulation factors, and the vascular endothelium. This “cellular model” of coagulation emphasizes that clot formation is not merely a series of enzymatic reactions, but a tightly regulated process occurring on cell surfaces and dependent on the complex interplay of cellular and plasma components (4). Together, these limitations underscore the need for assays that assess both the quantity and functional performance of hemostatic components under near-physiological conditions, providing a more representative evaluation of hemostasis *in vivo*.

Rotational thromboelastometry (ROTEM) offers a more comprehensive assessment of hemostasis by evaluating the entire hemostatic process in whole blood, from clot initiation to fibrinolysis. In this method, a citrated blood sample is placed in a preheated cup (37 °C) containing specific activating reagents, while an oscillating pin detects viscoelastic changes as the clot forms and lyses. The viscoelastic changes generated during clot formation are measured by the oscillating pin and subsequently converted into a tracing that provides a dynamic representation of coagulation kinetics. This mechanical measurement distinguishes ROTEM from other viscoelastographic systems, which record and display the signal directly as it is detected. Key parameters including clotting time (CT), clot formation time (CFT), alpha angle (*α*), amplitude (A), maximum clot firmness (MCF), and lysis indices (LI, ML) provide detailed insights into clot formation, strength, and dissolution. Unlike plasma-based assays, ROTEM captures the interactions among red and white blood cells, platelets, and plasma coagulation factors under near-physiological conditions, providing a more representative assessment of *in vivo* hemostasis (5, 6).

Initially developed for human medicine, ROTEM has since been applied in veterinary settings for diagnosing bleeding disorders, perioperative monitoring, and guiding transfusion therapy (5–11). The ROTEM Sigma analyzer represents a significant evolution over the previous Delta system, primarily due to fully automated cartridges and an integrated sample handler. Both systems use the same pin-and-cup technology, but Sigma cartridges contain lyophilized reagents, allowing four simultaneous tests with minimal manual intervention. The procedure consists of simply inserting the cartridge and attaching the blood sample, thus minimizing pipetting errors, preanalytical variability, and operator dependence. These improvements enhance overall analytical reliability and biosafety, optimize workflow efficiency, particularly in the emergency and intensive care settings, and minimize the risk of sample contamination, thereby supporting faster and more accurate diagnosis and treatment for acutely bleeding patients (12).

Although the ROTEM Sigma system offers significant improvements in automation and consistency, reference intervals established for the Delta system may not be directly applicable. Nevertheless, human studies have demonstrated good overall agreement between the two analyzers, with most parameters yielding comparable results, despite minor differences for certain parameters such as clotting time and FIBTEM values (13–17).

As ROTEM technology becomes increasingly accessible in veterinary medicine and clinical interest continues to grow, reference data remains largely limited to the earlier Delta system (18–20). Species-specific reference intervals have already been established for dogs using the Delta platform, but no equivalent data is yet available for the Sigma analyzer. Given its simplified operation and extensive automation, the Sigma system is expected to become more widely adopted in clinical practice than the Delta. Establishing Sigma-specific reference intervals is therefore essential to ensure accurate clinical interpretation and to evaluate the comparability of results between analyzers. Such comparison is crucial for determining whether data generated by the two systems can be used interchangeably, thereby supporting multicentric studies, cross-platform harmonization, and longitudinal monitoring across institutions.

Therefore, this study aimed to establish reference values for ROTEM Sigma in healthy dogs, compare these values with previously reported data from the Delta system in order to assess their potential interchangeability, and to evaluate the potential influence of biological variables.

## 2 Materials and methods

### 2.1 Animal enrollment

This prospective observational study enrolled dogs deemed clinically healthy based on complete medical history, physical examination, and hematological assessment performed using a ProCyte Dx hematology analyzer (IDEXX Laboratories, Westbrook, ME, United States). Inclusion criteria required normal hematologic values and absence of clinical illness. Exclusion criteria included hematologic abnormalities, specifically hematocrit values outside the analyzer’s established reference interval of 37.3–61.7% (i.e., anemia or polycythemia) and platelet counts outside 148–484 K/μL (i.e., thrombocytopenia or thrombocytosis). Dogs that had received medications known to interfere with coagulation within the previous month, such as nonsteroidal anti-inflammatory drugs (e.g., acetylsalicylic acid), antithrombotic agents (clopidogrel, heparin, rivaroxaban), or rodenticides (21), were also excluded.

A minimum sample size of 40 patients was selected in accordance with the American Society for Veterinary Clinical Pathology (ASVCP) reference interval guidelines, which recommend this number to ensure accurate calculation of the 2.5–97.5th percentiles and reliable statistical performance in small cohorts (22, 23).

All dogs included in the study were either regular blood donors, privately owned by hospital staff, or patients presented for routine orthopedic consultations who were not receiving any active treatment. Blood samples for ROTEM analysis were collected from conscious unsedated, gently restrained dogs at the same time as routine hematological testing performed as part of the animals’ standard medical care, thereby avoiding any additional venipuncture or procedures beyond standard clinical care. Owners’ consent was obtained for all patients prior to enrolment.

### 2.2 Sample collection

Whole blood was collected from either the external saphenous or jugular vein after disinfecting the skin with 70% isopropyl alcohol, following standard in-house procedures. The venipuncture site was allowed to air dry before collection. For each dog, blood was collected using a sterile 23G needle attached to a 5 mL syringe. Samples were drawn directly into the syringe and transferred immediately into the appropriate tubes without exposure to any interim container, thereby minimizing contact activation. From this sample, 0.5 mL was placed into an EDTA tube for hematological analysis, and 2.7 mL was transferred into a 3.2% sodium citrated tube for ROTEM analysis. In accordance with the manufacturer’s recommendations, the citrated blood tube was filled through the cap under vacuum, maintaining appropriate negative pressure. The citrated sample was then homogenized on an agitator at room temperature (20–25 °C) for approximately 30 min before analysis to ensure stability and uniformity.

### 2.3 ROTEM analysis

Thromboelastometry (ROTEM^®^ Sigma; Werfen TEM Innovations GmbH, Munich, Germany) was performed according to the manufacturer’s instructions using cartridges preloaded with lyophilized reagents for EXTEM, INTEM, FIBTEM, and HEPTEM assays. Prior to each analysis, the appropriate cartridge was removed from its protective packaging and inserted into the analyzer. The citrated blood collection tube was then clipped directly onto the sampling needle with the cap facing downward, allowing the needle to pierce the cap and automatically aspirate the required sample volume into the cartridge. Once the tube was in place, the analyzer automatically initiated the assay, reconstituting the lyophilized reagents and performing the measurements. Each analysis required 60 min to complete. Quality control procedures and routine maintenance were performed in strict accordance with the manufacturer’s recommendations.

Each assay cartridge contains specific activators or inhibitors tailored to isolate and evaluate different components of the coagulation process. EXTEM uses Tissue Factor as an activator to assess the extrinsic pathway of coagulation, focusing on fibrin polymerization and fibrinolysis, and is comparable to the Prothrombin Time assay (PT). INTEM employs Ellagic Acid and Phospholipids as activators to assess the intrinsic pathway, providing insights into fibrin polymerization and fibrinolysis, and is analogous to the Activated Partial Thromboplastin Time assay (aPTT). HEPTEM contains Ellagic Acid and Lyophilized Heparinase, allowing neutralization of heparin and evaluation of coagulation in case of heparin therapy. FIBTEM, derived from EXTEM, incorporates Cytochalasin D, a platelet inhibitor, to isolate fibrin polymerization by excluding platelet contributions to clot formation.

The ROTEM parameters assessing various aspects of coagulation are defined as follows.

- Clotting Time (CT): time from test initiation to the first detectable clot (amplitude of 2 mm) measured in seconds.
- Amplitude (A10): clot amplitude 10 min after CT, reflecting the early kinetic of clot formation, measured in millimeters.
- Maximal Clot Firmness (MCF): maximal clot strength, representing the combined effects of platelet activity, fibrin formation, and factor XIII cross-linking, measured in millimeters.
- Lysis Indices (LI60, ML): LI60 represents the percentage of clot firmness at 60 min, while ML (maximum lysis) quantifies the overall degree of fibrinolysis expressed as a percentage of MCF.
- Shear Modulus Strength (G): a calculated measure of mechanical clot strength (dyn/cm^2^), derived from the formula: G = (5,000 × MCF)/(100−MCF). Elevated G values indicate hypercoagulability, whereas reduced G values indicate hypocoagulability (24–29).

### 2.4 Data collection

Demographic data (age, sex, weight, neutered status and breed), hematologic parameters, and ROTEM Sigma results were collected for each dog. All data were recorded at the time of sample analysis by the same operator to ensure consistency. ROTEM results were directly exported from the analyzer software into a standardized Excel spreadsheet, while hematologic data were retrieved from the hospital laboratory information system and compiled within the same file.

### 2.5 Statistical analysis

Data was first assessed for normality using the Shapiro–Wilk test. For normally distributed variables, results were expressed as mean ± standard deviation (SD) with 95% confidence intervals (95% CI). Comparisons with previously reported values or theoretical means were performed using an independent t-test. For non-normally distributed variables, median and interquartile range (IQR) were reported, and comparisons were conducted using the Mann–Whitney U test. Comparisons with previously reported values or theoretical means were performed using an independent t-test.

Continuous ROTEM parameters (CT, A10, MCF, ML, LI60, and G-calculated) were initially summarized using the classical parametric methods to establish preliminary reference intervals before applying the robust method for comparison. For each parameter and assay (EXTEM, INTEM, HEPTEM and FIBTEM), the mean and standard deviation were computed across all healthy adult canine samples. Reference limits were then defined as mean ± 1.96 × SD, providing an approximate 95% coverage under the assumption of normality.

Given the potential influence of skewness and outliers in coagulation data, the same parameters were subsequently analyzed using Horn’s robust median–MAD method (30). Median and MAD values were iteratively recalculated after excluding observations beyond ±3.5 × MAD until stabilization, and the robust standard deviation was estimated as MAD × 1.4826, and reference limits were then defined as median ± 1.96 × (1.4826 × MAD). Confidence intervals were derived using a nonparametric bootstrap approach.

Both conventional and robust reference intervals were compared with those reported in three published studies (18–20), which provided the most comprehensive set of ROTEM Delta outcomes. Percent overlap was calculated between our intervals and each published 2.5–97.5th percentile range, defined as the length of the intersection divided by the published span. Although no formal reference defines a threshold for clinically significant divergence, intervals with less than 50% overlap are considered to represent a meaningful difference, as this indicates that at least half of the values are not shared between datasets, which may be clinically relevant. Concordance across assays was visualized using a faceted forest plot with separate panels for EXTEM, INTEM, and FIBTEM, displaying our median–MAD intervals alongside published intervals. Comparison with HEPTEM could not be performed because no published reference intervals were available for this assay.

Multivariate analysis was performed to assess the effects of demographic factors (age, sex, neuter status) and hematologic variables on ROTEM parameters. Linear regression model was applied to continuous ROTEM parameters, whereas logistic regression was used when appropriate for categorical outcomes. Variables with a *p*-value < 0.2 in univariate analysis were included in the multivariate model. Statistical significance was set at *p* < 0.05.

## 3 Results

### 3.1 Demographic results

The study population included 42 dogs. Mixed-breed dogs were the most common (n = 6), followed by Golden Retrievers (n = 4), Staffordshire Bull Terriers (n = 4), and American Bullies (n = 3). Other breeds were represented by one or two individuals, including French Bulldogs and Belgian Malinois (n = 2 each), and Australian Shepherd, Beagle, Beauceron, Bernese Mountain Dog, Cocker Spaniel, Dachshund, English Bulldog, German Shepherd, Husky, Jack Russell Terrier, Newfoundland, Poodle, Pug, Samoyed, Shih Tzu, Spitz, Swiss Shepherd, Weimaraner, Welsh Corgi Pembroke, West Highland White Terrier, and Yorkshire Terrier (n = 1 each).

Of the 42 dogs included, 16 were males (38%) and 26 were females (62%). A total of 10 dogs (24%) were intact, with an equal distribution between males and females (n = 5 each), while the remaining 32 dogs (76%) were neutered. This corresponded to a neutering rate of 69% in males and 81% in females.

The median age was 4.8 years (min 0.6, max 15 years; interquartile range [IQR]: 3.6–7.6 years). The median body weight was 23.3 kg (min 5, max 60 kg; [IQR]: 11.2–32.9 kg).

### 3.2 ROTEM Sigma reference intervals

Reference intervals for ROTEM Sigma parameters (INTEM, EXTEM, FIBTEM, and HEPTEM) were calculated using both conventional parametric and robust statistical methods and are summarized in Table 1.

**Table 1:** Reference intervals for ROTEM Sigma parameters (EXTEM, INTEM, FIBTEM, and HEPTEM) calculated using conventional parametric and robust methods in dogs.

Parameter	EXTEM		INTEM		FIBTEM		HEPTEM	
	Classic	Robust	Classic	Robust	Classic	Robust	Classic	Robust
CT (s)	[39.9–54.9]	[25.58–64.42]	[111.4–143.1]	[98.85–151.15]	–	–	[41.9–216.3]	[92.13–161.87]
MCF (mm)	[44.9–67.1]	[35.80–72.20]	[54.1–61.3]	[44.38–67.62]	[4.8–6.7]	[1.09–6.91]	[33.1–82.3]	[45.38–68.62]
A10 (mm)	[29.9–40.7]	[25.02–52.98]	[36.8–44.8]	[29.47–58.53]	[4.1–5.7]	[1.09–6.91]	[15.5–64.7]	[24.56–59.44]
ML (%)	[−1.1–21.9]	[−3.00–35.00]	[2.9–13.7]	[−1.72–15.72]	[−1.0–1.8]	[0.00–0.00]	[0–47.5]	[−2.62–20.62]
LI60 (%)	[100–100]	[100.00–100.00]	[97.7–100.6]	[100.00–100.00]	–	[100.00–100.00]	[93.6–100]	[100.00–100.00]
G	[5783.9–8374.3]	[3941.47–10861.11]	[6245.7–7937.5]	[3938.87–9590.43]	[251–360]	[117.66–408.74]	[0–13,922]	[3355.93–9141.57]

### 3.3 Comparison with ROTEM Delta reference values

Comparisons between the ROTEM Sigma values obtained in this study and those previously reported for the ROTEM Delta model (18–20) were summarized for each ROTEM profile.

**Figure 1:**
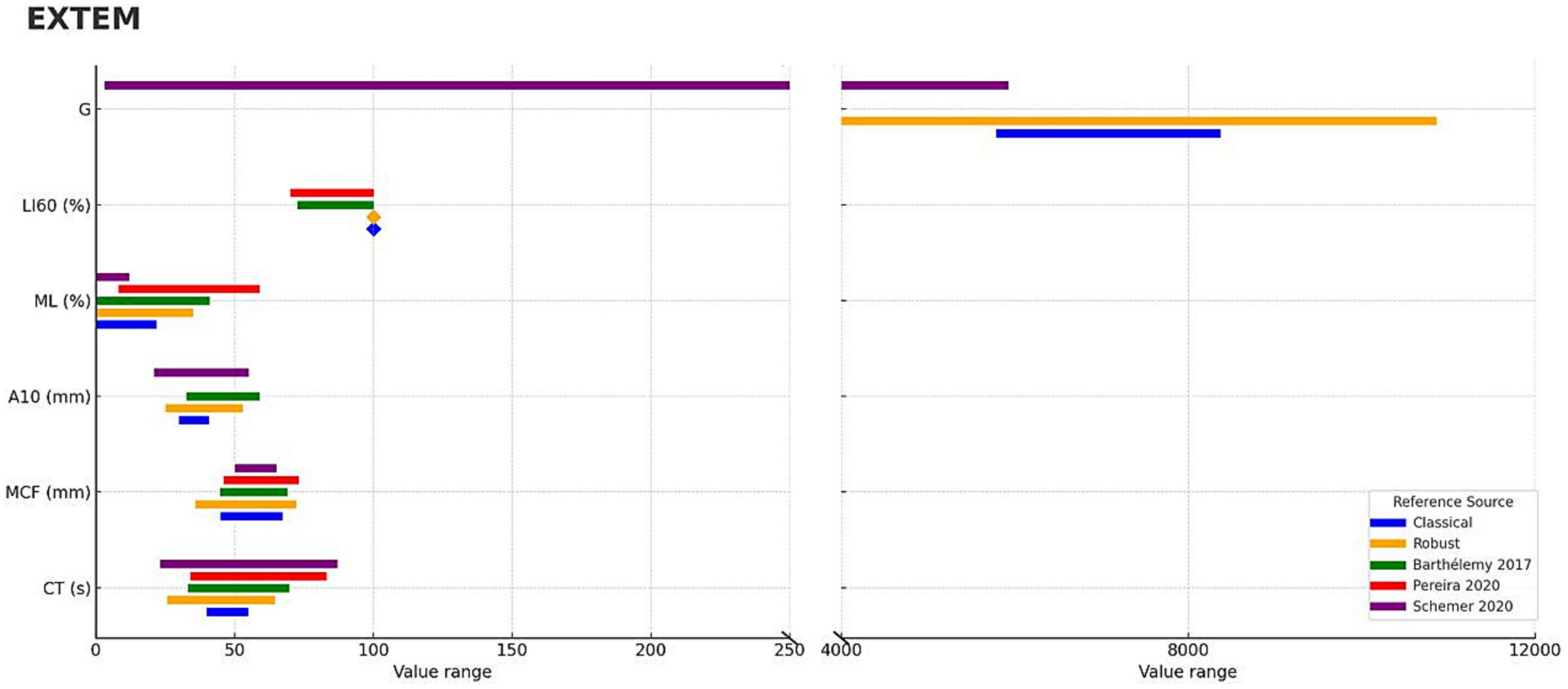
Concordance of ROTEM Sigma, EXTEM parameters compared with previously published studies using ROTEM Delta in dogs. Continuous ROTEM parameters (CT, MCF, A10, ML, LI60, G) are shown for EXTEM panels. Blue bars represent classical reference intervals derived from this study, while orange bars represent robust median–MAD intervals. Diamond markers are used to display a value when the range is too narrow or when a single constant value needs to be shown. Published reference intervals from three studies are shown in green (18), red (19), and purple (20) bars.

For the EXTEM assay, the degree of overlap between reference intervals reported in the literature varied across parameters (Table 2) with a visual representation provided for clarity (Figure 1). The CT showed a partial overlap ranging from 60.7 to 85.6% between studies. The MCF exhibited a high level of consistency, with overlap values between 97.0 and 100.0%. For A10, the overlap ranged from 77.2 to 82.2%, indicating moderate agreement between intervals. The ML parameter showed greater variability, with overlap values ranging from 52.9 to 100.0% depending on the study. In contrast, LI60 showed complete concordance (100%) across the available data, suggesting a ceiling effect. Finally, for the G parameter, the observed overlap was more limited, at approximately 33%.

**Table 2:** Robust reference intervals and overlap percentages for ROTEM Sigma EXTEM parameters in dogs, compared with classical reference intervals previously published for ROTEM Delta.

EXTEM							
Parameter	Robust model	Barthélemy et al. (18)		Pereira et al. (19)		Schefer et al. (20)	
		Reference value	Overlap	Reference value	Overlap	Reference value	Overlap
CT (s)	[25.58–64.42]	[33.00–69.70]	85.60%	[34.00–83.00]	62.10%	[23.00–87.00]	60.70%
MCF (mm)	[35.80–72.20]	[44.60–69.00]	100.00%	[46.00–73.00]	97.00%	[50.00–65.00]	100.00%
A10 (mm)	[25.02–52.98]	[32.59–59.00]	77.20%	–	–	[21.00–55.00]	82.20%
ML (%)	[−3.00–35.00]	[0.00–41.00]	85.40%	[8.00–59.00]	52.90%	[0.00–12.00]	100.00%
LI60 (%)	[100.00–100.00]	[72.60–100.00]	100.00%	[70.00–100.00]	100.00%	–	–
G (dyn·s)	[3941.47–10861.11]	–	–	–	–	[3–5,928]	33.00%

**Figure 2:**
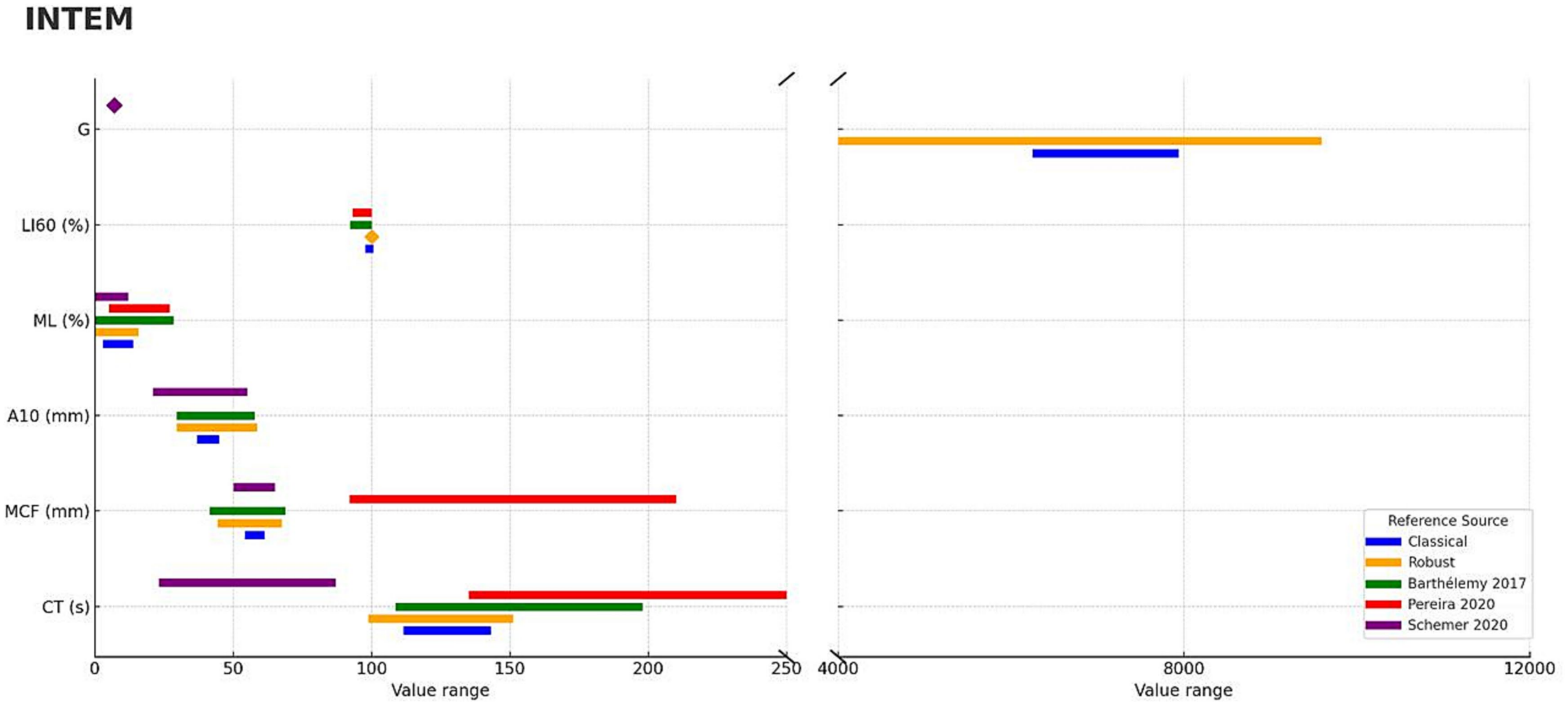
Concordance of ROTEM Sigma, INTEM parameters compared with previously published studies using ROTEM Delta in dogs. Continuous ROTEM parameters (CT, MCF, A10, ML, LI60, G) are shown for INTEM panels. Blue bars represent classical reference intervals derived from this study, while orange bars represent robust median–MAD intervals. Diamond markers are used to display a value when the range is too narrow or when a single constant value needs to be shown. Published reference intervals from three studies are shown in green (18), red (19), and purple (20) bars.

For the INTEM assay, systematic differences were observed when compared with previously published reference intervals (Table 3), with these discrepancies visually summarized (Figure 2). The degree of overlap varied across parameters, ranging from 0.0 to 100.0%. The CT parameter showed limited agreement between studies, with overlap values ranging from 0.0 to 47.6%. The MCF exhibited variable concordance, with percentage overlap ranging from 0.0 to 100.0% depending on the study. For A10, the overlap ranged from 75.1 to 100.0%, indicating moderate consistency between reference ranges. The ML parameter also displayed variable overlaps, from 48.7 to 100.0%. In contrast, LI60 showed complete overlap (100.0%) across the available datasets, suggesting a possible ceiling effect. Finally, the overlap for the G parameter was minimal, estimated at 0.0%.

**Table 3:** Robust reference intervals and overlap percentages for ROTEM Sigma INTEM parameters in dogs, compared with classical reference intervals previously published for ROTEM Delta.

INTEM							
Parameter	Robust	Barthélemy et al. (18)		Pereira et al. (19)		Schefer et al. (20)	
		Reference value	Overlap	Reference value	Overlap	Reference value	Overlap
CT (s)	[98.85–151.15]	[108.58–198.00]	47.60%	[135.00–283.00]	10.90%	[23.00–87.00]	0.00%
MCF (mm)	[44.38–67.62]	[41.50–68.70]	85.50%	[92.00–210.00]	0.00%	[50.00–65.00]	100.00%
A10 (mm)	[29.47–58.53]	[29.50–57.70]	100.00%	–	–	[21.00–55.00]	75.10%
ML (%)	[−1.72–15.72]	[0.00–28.40]	55.40%	[5.00–27.00]	48.70%	[0.00–12.00]	100.00%
LI60 (%)	[100.00–100.00]	[92.20–100.00]	100.00%	[93.00–100.00]	100.00%	–	–
G (dyn·s)	[3938.87–9590.43]	–	–	–	–	[7–9]	0.00%

**Figure 3:**
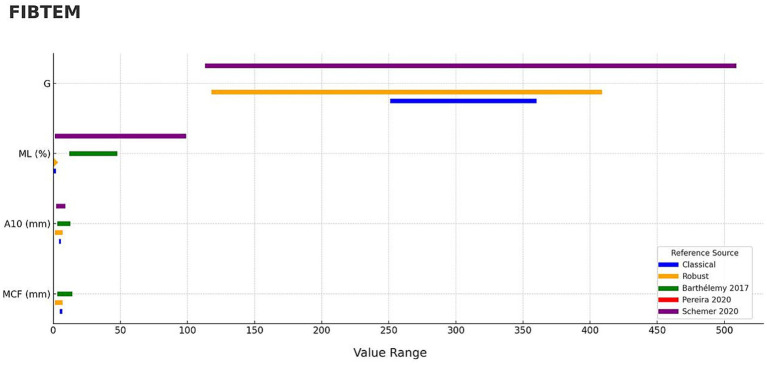
Concordance of ROTEM Sigma, FIBTEM parameters compared with previously published studies using ROTEM Delta in dogs. Continuous ROTEM parameters (MCF, A10, ML, G) are shown for FIBTEM panels. Blue bars represent classical reference intervals derived from this study, while orange bars represent robust median–MAD intervals. Diamond markers are used to display a value when the range is too narrow or when a single constant value needs to be shown. Published reference intervals from three studies are shown in green (18), red (19), and purple (20) bars.

For the FIBTEM assay, the degree of concordance between reference intervals reported in the literature was generally low (Table 4), as shown graphically (Figure 3). The MCF parameter showed limited overlap, estimated at 17.1%. For A10, the overlap ranged from 16.5 to 70.1%, indicating variable agreement across studies. The ML parameter showed no overlap (0.0%) with previously published intervals. In contrast, the G parameter demonstrated a higher level of concordance, with percentage overlap of 73.5%.

**Table 4:** Robust reference intervals and overlap percentages for ROTEM Sigma FIBTEM parameters in dogs, compared with classical reference intervals previously published for ROTEM Delta.

FIBTEM							
Parameter	Robust	Barthélemy et al. (18)		Pereira et al. (19)		Schefer et al. (20)	
		Reference value	Overlap	Reference value	Overlap	Reference value	Overlap
MCF (mm)	[1.09–6.91]	[3.00–14.00]	17.10%	–	–	–	–
A10 (mm)	[1.09–6.91]	[3.00–12.70]	16.50%	–	–	[2.00–9.00]	70.10%
ML (%)	[0.00–0.00]	[11.90–47.70]	0.00%	–	–	[1.00–99.00]	0.00%
G (dyn·s)	[117.66–408.74]	–	–	–	–	[113.00–509.00]	73.50%

Overall, these comparisons indicate that while EXTEM values are generally consistent with previous reports, INTEM and FIBTEM parameters differ substantially, highlighting the need for instrument-specific reference intervals.

### 3.4 Determinants of ROTEM Sigma parameters

**Figure 4:**
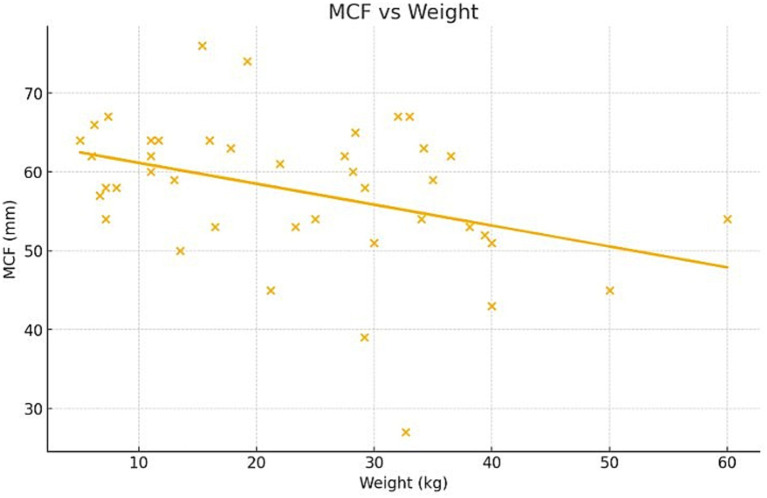
Relationship between body weight and maximum clot firmness (MCF) as measured by ROTEM Sigma in healthy dogs. Scatter plot showing a negative association between body weight and MCF (*p* = 0.021).

**Figure 5:**
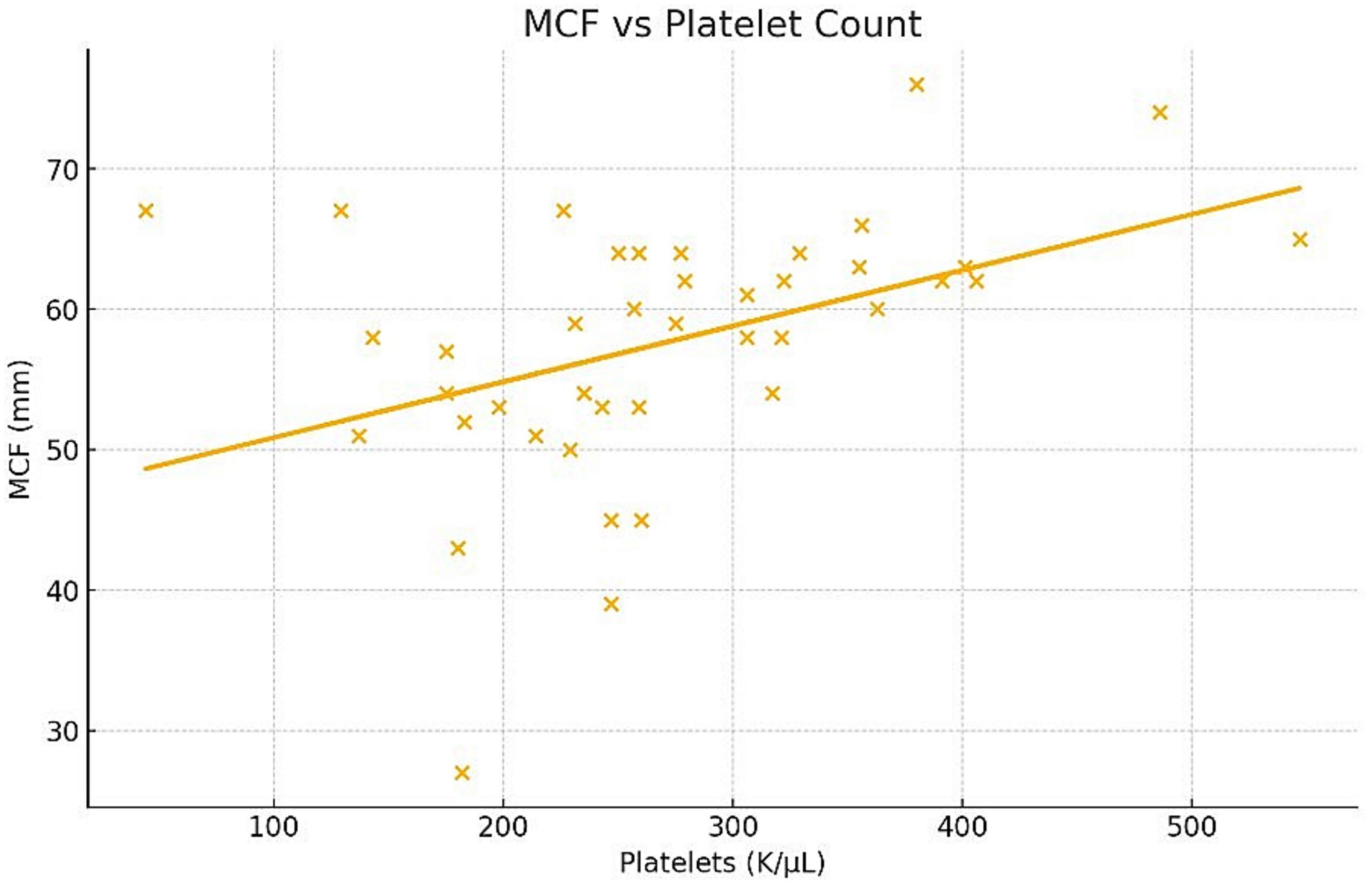
Relationship between platelet count and maximum clot firmness (MCF) as measured by ROTEM Sigma in healthy dogs. Scatter plot illustrating the positive correlation between platelet count and MCF (*p* = 0.0068).

**Figure 6:**
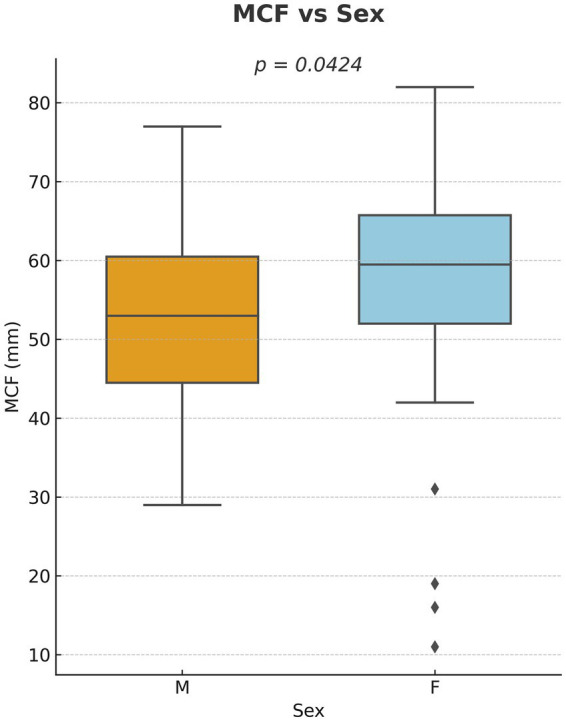
Influence of sex on maximum clot firmness (MCF) as measured by ROTEM Sigma in healthy dogs. Boxplot comparing MCF values between male and female dogs. A statistically significant difference was observed (*p* = 0.0424).

Multivariate regression revealed significant associations between maximum clot firmness (MCF) and several variables. Specifically, MCF decreased by 0.25 mm for each additional kilogram of body weight (*p* = 0.021) (Figure 4) and increased by 0.041 mm for every 1,000 platelets/μL (*p* = 0.0068) (Figure 5). A minor but statistically significant effect of sex (*p* = 0.0424) was also observed, with males exhibiting a mean MCF approximately 2 mm lower than that of females (Figure 6). No significant effect of age, hematocrit, or neuter status was detected.

## 4 Discussion

This study provides a comprehensive set of reference intervals for canine blood using the ROTEM Sigma analyzer, including EXTEM, INTEM, FIBTEM, and HEPTEM, with the establishment of reference intervals for HEPTEM being a particularly novel aspect of this work, as no prior Sigma or Delta data have been published in dogs. Reference intervals were calculated using both classical parametric and robust median–MAD approaches. The latter following Horn’s recommendations for improved reliability in the presence of skewed or non-Gaussian data. The robust method is particularly relevant for viscoelastic parameters that may approach upper analytical limits (e.g., values capped at 100%), as such truncation can distort normality and inflate standard deviation–based estimates. By reducing the influence of outliers and bounded data, the robust approach yields intervals that more accurately reflect the central tendency of the population and are therefore more clinically meaningful. Importantly, previously published ROTEM Delta reference intervals used for comparison in this study are derived from a classical statistical approach rather than a robust model, introducing a methodological difference that should be considered when interpreting cross-platform comparisons. Although direct comparison with Delta data is not possible for HEPTEM, these newly established values lay the groundwork for future cross-platform evaluations. Alpha angle and CFT parameters are not available on the Sigma platform, limiting direct comparability with previously published Delta data, but highlighting the importance of Sigma-specific reference standards.

Overall, results demonstrated strong concordance between ROTEM Sigma and previously published Delta data, particularly for EXTEM parameters, indicating that extrinsic pathway activation is minimally affected by analyzer differences. In contrast, INTEM and FIBTEM exhibited greater variability, consistent with human studies (12–17), likely reflecting differences in assay design, reagent formulation, and intrinsic pathway or fibrin polymerization kinetics. These findings highlight the necessity of establishing analyzer-specific reference intervals to ensure accurate clinical interpretation while confirming that ROTEM Sigma provides results broadly comparable to Delta for EXTEM assays. They also underscore the importance of reporting analyzer-specific data in future clinical studies that include ROTEM results.

Extending these assay-specific observations, our study also explored demographic and biological influences on ROTEM parameters. Multivariate regression analyses indicated that body weight, platelet count, and sex significantly affected maximum clot firmness (MCF), highlighting additional sources of variability that are relevant for the clinical interpretation of Sigma-derived reference intervals.

The observed negative association between body weight and MCF suggests that larger dogs may exhibit slightly reduced clot firmness. However, this relationship is not well established in veterinary medicine, as the effect of body size on coagulation has rarely been specifically investigated. To our knowledge, only a single veterinary study has explicitly examined the impact of body size on ROTEM Delta parameters in healthy dogs (19). In that study, no statistically significant differences were observed between groups of dogs of varying weight, suggesting that body weight may have only modest or negligible effects on ROTEM measurements in broadly healthy canine populations. Interestingly, the relationship appears opposite in human medicine, where increased body weight and obesity are consistently associated with hypercoagulability (31, 32). Several factors could explain this discrepancy. First, unlike in humans, canine body weight does not necessarily reflect body condition score. In dogs, body weight is largely determined by breed, meaning that higher value more often indicates larger body size rather than excess body fat. Although all dogs in this study were clinically healthy and in good general condition, no formal body condition score (BCS) was recorded, preventing distinction between lean and overweight individuals. In human medicine, normalization to height-predicted (ideal) body weight is commonly used to differentiate the effects of body size from those of adiposity. Similarly, incorporating BCS assessments within dogs of the same breed could help refine future analyses and clarify the influence of body composition on coagulation parameters. Second, breed-related variations in hematologic or platelet function may confound weight-related effects. For instance, Greyhounds and other sighthound breeds have been shown to exhibit relative hypocoagulability compared with other breeds of similar body weight, illustrating how breed-specific hemostatic profiles could influence ROTEM results independently of size (33, 34).

A positive correlation between platelet count and MCF was observed, which is physiologically expected given the central role of platelets in clot formation and firmness. Platelets provide structural support for fibrin polymerization, so higher platelet numbers naturally lead to stronger viscoelastic clot profiles. This result aligns with previous canine studies consistently identifying platelet count as a key determinant of clot strength across both TEG and ROTEM analyses (2, 20, 35, 36), reinforcing the importance of interpreting viscoelastic data in conjunction with hematologic parameters.

Although sex showed a statistically significant effect on MCF in our cohort, the magnitude of this effect was small and likely of limited clinical relevance. Previous veterinary studies have similarly reported only minor or inconsistent differences in viscoelastic parameters related to sex (4, 18, 19, 37, 38). Some evidence even suggests that reproductive status (particularly neutering) may exert a greater influence than sex itself, with spayed females occasionally showing higher clot firmness (19). In human medicine, females are frequently reported to display a more hypercoagulable profile than males (39, 40), likely driven by hormonal influences. In our cohort, the estrous cycle stage of intact females was not recorded, which could have masked potential fluctuations. In diseased populations, sex-related effects might be more pronounced, as inflammation or metabolic changes can enhance physiological differences. Altogether, inconsistencies across studies likely arise from variations in population structure, reproductive status distribution, and methodological factors such as activator choice and the instrument used.

No significant associations were observed between age, hematocrit, or neuter status and ROTEM parameters in our cohort. This contrasts with a single previous study (18) reporting age-related hypercoagulability in healthy dogs, characterized by faster clot initiation (shorter CT) and increased clot firmness (higher MCF), findings that are consistent with observations in humans (41, 42). The absence of age-related differences in our study may be explained by the age distribution of our cohort, which included few older dogs, as well as the exclusion of individuals with comorbidities or chronic conditions that could independently influence coagulation. Hematocrit was not associated with ROTEM parameters, likely because most dogs had values within the normal reference range and anemic individuals were excluded. As such, our population did not allow evaluation of the effects of anemia on viscoelastic profiles. Within the physiological range observed, hematocrit did not appear to influence coagulation dynamics. In contrast, previous studies have shown that clinical anemia can promote hypercoagulability even in otherwise healthy dogs (43–45). Finally, neuter status did not appear to influence ROTEM parameters in our cohort. This may be due to the relatively balanced distribution of reproductive status among dogs or the limited sample size, which was likely insufficient to detect any potential differences.

While the study provides valuable baseline data for ROTEM Sigma in dogs, some limitations should be detailed. First, the study population size (n = 42) was relatively small, although consistent with ASVCP recommendations for reference interval determination using robust statistical methods (22). Larger multicentric datasets would improve precision and allow breed-specific reference intervals to be established. Second, although sampling conditions were standardized, the site of venipuncture (jugular vs. saphenous) might have influenced coagulation activation. According to PROVETS guidelines (23), the impact of the vein used is more related to vessel size than to the specific vessel itself. In our study, the population was predominantly composed of large dogs, meaning that both jugular and saphenous veins generally provided large, free-flowing vessels, and thus the site of venipuncture likely had minimal impact on coagulation. Additionally, blood was collected immediately after skin puncture with minimal aspiration and careful handling, thereby minimizing preanalytical variability. Furthermore, although a consistent needle gauge (23G) was used in all dogs to standardize phlebotomy procedures, PROVETS guidelines (23) suggest that larger-bore needles (≥21G) may further reduce flow-induced shear stress; thus, the use of a 23G needle represents a potential preanalytical limitation that cannot be completely excluded. Notably, in human pediatric practice, 23G needles are commonly used to limit pain and reduce hemolysis during blood collection (46), particularly for delicate or small veins. Similarly, using a 23G needle in small dogs allowed safe and consistent sampling while minimizing the risk of pre-analytical coagulation activation. Third, the study population consisted exclusively of dogs considered clinically healthy at enrollment, including regular blood donors, staff-owned pets, and patients presented for routine orthopedic consultations without concurrent medical treatment. Although no explicit exclusion criteria based on recent illness or inflammatory status were applied, this selection was designed to minimize confounding effects of inflammation on ROTEM measurements. Blood samples were collected prior to any surgical intervention, ensuring assessment of baseline hemostatic status. Orthopedic patients may have had localized musculoskeletal inflammation; however, in the absence of systemic illness or active therapy, such inflammation is unlikely to meaningfully influence coagulation. This approach is consistent with previous veterinary studies showing that dogs presenting for orthopedic consultations generally exhibit normal coagulation profiles prior to surgery (47). Nevertheless, the potential influence of subclinical or undetected inflammation on ROTEM results cannot be entirely excluded. Finally, it should be noted that direct comparative validation between the Sigma and Delta systems using paired samples was not performed in the present study, representing an additional limitation and an important avenue for future research. Such comparative studies would be valuable to further clarify analytical equivalence and support the broader integration of Sigma-based viscoelastic testing in veterinary medicine.

While our study establishes robust reference intervals for ROTEM Sigma, it is also important to contextualize these findings relative to the established ROTEM Delta system, which remains widely used in veterinary practice. ROTEM Sigma offers a fully automated, user-friendly platform with reduced hands-on time, minimized operator-dependent variability, and built-in quality control, supporting consistent performance even with less-experienced staff or multiple assays run simultaneously. Regarding sample volume, ROTEM Sigma requires a standard 2.7 mL citrated tube per analysis, as recommended by the manufacturer. Remaining blood after testing can be used for additional analyses, and while smaller tubes could be considered for very small patients, the volume is generally manageable. In comparison, ROTEM Delta requires ~300–340 μL per cartridge but performing a full four-assay panel totals ~1.6 mL, not including losses due to pipetting, narrowing the practical difference to roughly 1 mL. Thus, despite ROTEM Delta’s lower per-cartridge volume, ROTEM Sigma’s automation, reduced manual error, and integrated QC provide clear operational advantages. Overall, ROTEM Sigma combines automation, reliability, and operational simplicity, making it highly suitable for routine clinical use, while reference intervals specific to this platform ensure accurate interpretation.

The study is not designed to validate ROTEM Sigma against Delta data. The comparisons presented are purely descriptive and discussion-based, aiming to contextualize Sigma reference intervals within previously published Delta results and illustrate potential platform differences. Direct comparison of ROTEM Sigma and Delta is inappropriate for establishing equivalence due to differences in methodology, measurement mechanisms, sample handling, and the dynamic nature of hemostatic profiles. Demonstrating true method comparability would require paired, simultaneous testing on both analyzers with Bland–Altman analysis of agreement, which falls outside the scope of this work. Substantial variability exists even among ROTEM Delta studies, reflecting methodological and population differences. This underscores that reference intervals are platform-specific and should not be assumed interchangeable. These comparisons emphasize the importance of establishing and applying analyzer-specific reference intervals for accurate clinical interpretation.

## 5 Conclusion

This study provides both classical and robust reference intervals for ROTEM Sigma in clinically healthy dogs, establishing essential analyzer-specific baselines for veterinary viscoelastic testing. EXTEM parameters showed close agreement with previously published Delta data suggesting similar clinical applicability between platforms, whereas the greater variability observed for INTEM and FIBTEM assays supports cautious interpretation in clinical comparisons. Maximum clot firmness (MCF) appeared to be influenced by body weight, platelet count, and sex, whereas other parameters had minimal impact. Importantly, the reference intervals generated using robust statistical methods are recommended for clinical use, providing validated, species-specific baselines for interpretation of ROTEM results in veterinary patients. As viscoelastic testing continues to expand in veterinary medicine, the availability of simpler, semi-automated systems such as the Sigma may facilitate its broader clinical use—provided that validated, species-specific reference intervals like those established here are available.

## Data Availability

The raw data supporting the conclusions of this article will be made available by the authors, without undue reservation.
