# Correction: Determination of canine ROTEM Sigma reference ranges and comparative analysis with ROTEM Delta

**DOI:** 10.3389/fvets.2026.1832992

**Published:** 2026-04-07

**Authors:** Aurélie Jourdan, Julia Noval Montero, Geoffrey Troussier, Anna Anderco, Caroline Dania, Yann Moumadah, Maxime Cambournac

**Affiliations:** 1 Centre Hospitalier Vétérinaire Frégis, Paris, France; 2 IVC Evidensia France, Courbevoie, France

**Keywords:** clot strength, coagulation, hemostasis, reference intervals, rotational thromboelastometry, ROTEM, viscoelastic testing

In the published article, the DOIs for eight of the listed citations were incorrect and pointed to different articles than those cited in the text.

These errors are strictly bibliographical and do not affect the data, results, or conclusions of our study, and have been rectified as follows:

Reference 1 was erroneously written as Wiinberg B, Jensen AL, Johansson P, Rozanski E, Tranholm M, Kristensen AT. Thromboelastographic evaluation of hemostatic function in dogs with disseminated intravascular coagulation. *J Vet Intern Med*. (2008) 22:357–65. doi: 10.1111/j.1939-1676.2008.0043

It should be 1. Wiinberg B, Jensen AL, Johansson P, Rozanski E, Tranholm M, Kristensen AT. Thromboelastographic evaluation of hemostatic function in dogs with disseminated intravascular coagulation. *J Vet Intern Med*. (2008) 22:357–65. doi: 10.1111/j.1939-1676.2008.0058.x

Reference 9 was erroneously written as Conversy B, Blais MC, Dunn M, Gara-Boivin C, del Castillo JRE. Anticoagulant activity of oral rivaroxaban in healthy dogs. *Vet J*. (2017) 223:5–11. doi: 10.1016/j.tvjl.2017.04.003

It should be 9. Conversy B, Blais MC, Dunn M, Gara-Boivin C, del Castillo JRE. Anticoagulant activity of oral rivaroxaban in healthy dogs. *Vet J*. (2017) 223:5–11. doi: 10.1016/j.tvjl.2017.03.006

Reference 19 was erroneously written as Pereira JM, Silva AM, Silva MA. Reference intervals for rotational thromboelastometry in healthy dogs. *Vet J*. (2020) 253:105396. doi: 10.1016/j.tvjl.2019.09.022

It should be 19.Pereira JM, Silva AM, Silva MA. Reference intervals for rotational thromboelastometry in healthy dogs. *Vet J*. (2020) 253:105396. doi: 10.1016/j.rvsc.2020.01.019

Reference 28 was erroneously written as Weingand N, Vuille-Dit-Bille J, Jud Schefer R, Kutter APN, Stirn M, Adamik KN, et al. Evaluation of the effect of storage time on ROTEM^®^ S parameters in canine whole blood. *BMC Vet Res*. (2022) 18:1–8. doi: 10.1186/s13613-022-00989-3

It should be 28. Weingand N, Vuille-Dit-Bille J, Jud Schefer R, Kutter APN, Stirn M, Adamik KN, et al. Evaluation of the Effect of Storage Time on ROTEM S^®^ Parameters in Healthy and Ill Dogs. *BMC Vet Res*. (2022) 18:1–8. doi: 10.3390/ani12151996

Reference 32 was erroneously written as Bertaggia Calderara D, Aliotta A, Zermatten MG, Kröll D, Stirnimann G, Alberio L. Hyper-coagulability in obese patients accurately identified by combinations of global coagulation assay parameters. *Thromb Res*. (2020) 191:1–7. doi: 10.1016/j.th

It should be 32. Bertaggia Calderara D, Aliotta A, Zermatten MG, Kröll D, Stirnimann G, Alberio L. Hyper-coagulability in obese patients accurately identified by combinations of global coagulation assay parameters. *Thromb Res*. (2020) 191:1–7. doi: 10.1016/j.thromres.2020.01.012

Reference 35 was erroneously written as Turner JS, McKinley SK. Correlation of rotational thromboelastometry (ROTEM) parameters with platelet count and function in dogs. *Vet J*. (2019) 249:105396. doi: 10.1016/j.tvjl.2019.105396

It should be 35. Turner JS, McKinley SK. Correlation of rotational thromboelastometry (ROTEM) parameters with platelet count and their ability to predict thrombocytopenia in dogs. *Vet J*. (2019) 249:105396. doi: 10.1016/j.rvsc.2019.08.007

Reference 39 was erroneously written as Scarpelini S, Rhind SG, Nascimento B, Tien H, Shek PN, Peng HT, et al. Normal range values for thromboelastography in healthy adult volunteers. *Braz J Med Biol Res*. (2009) 42:1210–7. doi: 10.1590/S0100-879X2009001200009

It should be 39. Scarpelini S, Rhind SG, Nascimento B, Tien H, Shek PN, Peng HT, et al. Normal range values for thromboelastography in healthy adult volunteers. *Braz J Med Biol Res*. (2009) 42:1210–7. doi: 10.1590/s0100-879x2009001200015

Reference for 45 was erroneously written as Brooks AC, Guillaumin J, Cooper ES, Couto CG. Effects of hematocrit and red blood cell–independent viscosity on canine thromboelastographic tracings. *Vet Clin Pathol*. (2014) 43:154–63. doi: 10.1111/vcp.12127

It should be 45. Brooks AC, Guillaumin J, Cooper ES, Couto CG. Effects of hematocrit and red blood cell–independent viscosity on canine thromboelastographic tracings. *Vet Clin Pathol*. (2014) 43:154–63. doi: 10.1111/trf.12354

The original version of this article has been updated.

